# Protective Effect of Curcumin against Doxazosin- and Carvedilol-Induced Oxidative Stress in HepG2 Cells

**DOI:** 10.1155/2022/6085515

**Published:** 2022-02-11

**Authors:** Mariana Yazmin Medina-Pizaño, Marina Nayeli Medina-Rosales, Sandra Luz Martínez-Hernández, Liseth Rubi Aldaba-Muruato, José Roberto Macías-Pérez, Esperanza Sánchez-Alemán, Javier Ventura-Juárez, Martin Humberto Muñoz-Ortega

**Affiliations:** ^1^Center for Basic Sciences, Department of Morphology, Autonomous University of Aguascalientes, Aguascalientes, C.P. 20131, Mexico; ^2^Department of Clinical Chemistry, Faculty of Professional Studies Huasteca Zone, Autonomous University of San Luis Potosí, Ciudad Valles, San Luis Potosí 79060, Mexico; ^3^Center of Basic Sciences, Department of Chemistry, Autonomous University of Aguascalientes, 20131 Aguascalientes, Mexico

## Abstract

Doxazosin and carvedilol have been evaluated as an alternative treatment against chronic liver lesions and for their possible role during the regeneration of damage caused by liver fibrosis in a hamster model. However, these drugs have been reported to induce morphological changes in hepatocytes, affecting the recovery of liver parenchyma. The effects of these *α*/𝛽 adrenoblockers on the viability of hepatocytes are unknown. Herein, we demonstrate the protective effect of curcumin against the possible side effects of doxazosin and carvedilol, drugs with proven antifibrotic activity. After pretreatment with 1 *μ*M curcumin for 1 h, HepG2 cells were exposed to 0.1–25 *μ*M doxazosin or carvedilol for 24, 48, and 72 h. Cell viability was assessed using the MTT assay and SYTOX green staining. Morphological changes were detected using the hematoxylin and eosin (H&E) staining and scanning electron microscopy (SEM). An expression of apoptotic and oxidative stress markers was analyzed using reverse transcription-quantitative PCR (RT-qPCR). The results indicate that doxazosin decreases cell viability in a time- and dose-dependent manner, whereas carvedilol increases cell proliferation; however, curcumin increases or maintains cell viability. SEM and H&E staining provided evidence that doxazosin and carvedilol induced morphological changes in HepG2 cells, and curcumin protected against these effects, maintaining the morphology in 90% of treated cells. Furthermore, curcumin positively regulated the expression of *Nrf2*, *HO-1*, and *SOD1* mRNAs in cells treated with 0.1 and 0.5 *μ*M doxazosin. Moreover, the *Bcl-2*/*Bax* ratio was higher in cells that were treated with curcumin before doxazosin or carvedilol. The present study demonstrates that curcumin controls doxazosin- and carvedilol-induced cytotoxicity and morphological changes in HepG2 cells possibly by overexpression of *Nrf2*.

## 1. Introduction

The antihypertensive doxazosin, an *α*-1 adrenergic receptor blocker, has been used to treat benign prostatic hyperplasia and in trials of cardiovascular metabolic disorders as a lipid-lowering agent to prevent heart attack [1, 2]. In addition, doxazosin is known to induce apoptosis in prostate cancer cells [2]. Doxazosin has also been reported to induce apoptosis in cultured cardiomyocytes, possibly independently of blocking *α*-1 adrenergic receptors [1].

The apoptosis of cultured cardiomyocytes occurs dose dependently at concentrations ranging from 0.1 to 50 *μ*mol/L of doxazosin; this result was reported based on quinazoline, which reacts with different cell targets and leads to cell cycle arrest and, therefore, a decrease in cell proliferation [2]. In hypertensive patients, the serum doxazosin concentration reaches 0.122 and 0.244 *μ*mol/L with doxazosin doses of 8 and 16 mg, respectively [1].

Carvedilol (1-[carbazole-(4)-oxyl]-3-[(2-methoxy-phenoxyethyl)-amino]-2-propanol) is a nonselective *β*-adrenergic antagonist with vasodilator properties that shows an antagonist activity against the *α*-1 receptors. Additionally, carvedilol has been reported to act as a potent antioxidant [3].

A hamster liver cirrhosis model showed that treatment with doxazosin and carvedilol reduced the concentration of collagen fibers in the liver and improved liver parenchymal function with decreased AST and ALT levels. On one hand, the *α*-1 antagonist doxazosin modifies the typical morphology of hepatocytes and affects the regeneration process of the parenchyma [4]. On the other hand, the *β*-adrenoceptor blocker carvedilol is considered antifibrotic owing to its antioxidant activity demonstrated in a rat model of liver cirrhosis treated with carbon tetrachloride and an in vitro model of a liver stellar cell line (LX-2) [3, 5].

Curcumin is a bis-*α*, *β*-unsaturated *β*-diketone with antioxidant activity. The orthomethoxy group of curcumin plays an essential role in oxidative stress; it reacts with reactive oxygen species, and hydrogen donation reactions lead to oxidation and contribute to the well-established of reactive oxygen species- (ROS-) scavenging potential in biological systems [6].

Curcumin positively regulates cytoprotective and antioxidant proteins. However, it can induce a prooxidant effect at high doses [7]. One of the primary defense mechanisms against cytotoxicity is the stimulation of factor 2 related to erythroid nuclear factor 2 (*Nrf2*) and the activation of the antioxidant response element (ARE). Glutamate-cysteine ligase, thioredoxin reductase 1, NAD(P)H-quinone oxidoreductase 1, and heme oxygenase-1 (*HO-1*) are regulated by binding of *Nrf2* to this consensus binding sequence, *Nrf2* signaling pathway, and regulation of the expression of genes involved in ROS elimination [8].

In a previous study, the antifibrotic activity of doxazosin and carvedilol was demonstrated in a CCl_4_-induced liver fibrosis hamster model subjected to curcumin pretreatment; fibrosis was reversed by decreasing collagen I level, hepatocyte morphology remained intact, and the liver function was normal in the model. The antioxidant curcumin is proposed to increase the mRNA expression of *Nrf2*, whereas liver fibrosis decreases the antioxidant response of *Nrf2* and increases the expression of the nuclear factor kappa B (NF-*κ*B) pathway, a proinflammatory signaling pathway [9].

Based on the abovementioned reports, both doxazosin and carvedilol have been used experimentally to reduce liver fibrosis in vivo and were found to reduce collagen deposition and the number of cells with fibrogenic activity [4, 10, 11]. However, there is little information on the interaction of these drugs with hepatocytes. Therefore, in this study, we determined whether the interaction of these drugs with HepG2 cells as a model of liver cells causes cell damage, as well as evaluated the protective effect of curcumin against this damage.

## 2. Materials and Methods

### 2.1. Cell Culture

HepG2 cells (donated from the Immunoparasitology Laboratory, Universidad Autónoma de Aguascalientes, Mexico), were grown in DMEM (D5546; Sigma, St. Louis, MO, USA) supplemented with 2% fetal bovine serum (SU-420, microlab, Monterrey, NL, MEX), 2% L-glutamine (25-005-CI; Corning, NY, USA), and 1% penicillin/streptomycin (30-001-CI; Corning) at 37°C in a humidified atmosphere containing 5% CO_2_ and 95% O_2_, as proposed by ATCC. After incubation, the cells were rinsed with phosphate-buffered saline (PBS, pH 7.4). The cells were incubated with 1 mL of 0.25% trypsin EDTA for 7 min and harvested by trypsinization. The cells were then collected by centrifugation at 2,000 rpm for 5 min. All cells were harvested during the logarithmic phase of growth.

### 2.2. Treatments of the Cells with Doxazosin, Carvedilol, and Curcumin

Stock solutions of doxazosin (D9815; Sigma) and carvedilol (PHR1265; Sigma) were prepared at a concentration of 50 mM using DMSO as a solvent. Based on this solution, the preset treatments at concentrations of 0.1, 0.3, 0.5, 10, 15, and 25 *μ*M were obtained using complete DMEM as a solvent. A 1 mg/mL stock solution of curcumin (C1386; Sigma) in 1% DMSO was prepared. Subsequently, a dose-response curve was elaborated using successive dilutions of the stock solution (0.01, 0.03, 0.05, 0.07, 0.1, 0.5, 1, 5, 10, and 25 *μ*M/mL) and evaluating the viability of the HepG2 cells using the MTT assay, to apply 1 h before the interaction with doxazosin and carvedilol.

### 2.3. MTT Assay

HepG2 cells (1 × 10^4^ cells/well) were cultured in 96-well cell culture plates for 24 h prior to treatment and then treated with the concentrations (0.1, 0.3, 0.5, 10, 15, and 25 *μ*M) of doxazosin and carvedilol for 24, 48, and 72 h. This treatment was carried out with or without curcumin (1 *μ*M), and pretreatment with curcumin was carried out 1 h before exposure to doxazosin and carvedilol. Cell viability was measured using 5 mg/mL thiazolyl blue tetrazolium bromide (MTT) (M2128; Sigma) dissolved in PBS. Briefly, the cell culture medium with the treatment was changed to medium with diluted MTT (1 : 10, *v*/*v*) and incubated for 4 h at 37°C. After removing the incubation medium, formazan crystals were dissolved in 100 *μ*L of acid isopropanol (84 *μ*L of 25% HCl in 25 mL isopropanol). MTT reduction was quantified by measuring the light absorbance at 595 nm with a reference filter of 655 nm using a microplate reader spectrophotometer (Bio-Rad^®^ Laboratories, Hercules, CA, USA).

### 2.4. Hematoxylin and Eosin (H&E) Staining

Cells treated with doxazosin and carvedilol, with or without curcumin (1 *μ*M) pretreatment, were plated at 10^5^ cells per well on coverslips at the bottom of 24-well plates. After 24 h, the slide inside the plate was withdrawn and washed with 1x PBS; the cells were then fixed with 4% paraformaldehyde for 20 min at room temperature (25°C), washed with 1x PBS, stained with 500 *μ*L hematoxylin (1 min), followed by two washes with distilled water, counterstained with 500 *μ*L eosin for 20 s, followed by two washes with distilled water, and mounted in Mowiol^®^ (Sigma, 81381). The cells were visualized and analyzed at a magnification of 200x using a Axioskop 40/40 FL light microscope (Carl Zeiss AG, Germany) and analyzed using the Image-Pro Plus Software 4.5.1 (Media Cybernetics, Bethesda, MD, USA).

### 2.5. Scanning Electron Microscopy

HepG2 cells were analyzed by scanning electron microscopy to observe the morphological changes upon treatment with doxazosin and carvedilol, with or without curcumin (1 *μ*M) pretreatment, during the 24 h challenge. A total of 10^5^ cells grown on coverslips in 24-well plates were fixed in 2.5% glutaraldehyde for 10 min at 25°C. The samples were washed with distilled water and dehydrated before being placed on a critical point dryer for 30 min (IR Chamber Scope). Then, the mounted of the sample was carried out. Finally, the samples were plated with gold in Denton Vacuum before observing them with a scanning electron microscope (JEOL JSM-5900 Low Vacuum SEM, Japan).

### 2.6. SYTOX Green Staining

After completing the treatment with doxazosin and carvedilol, with or without curcumin (1 *μ*M) pretreatment, the DMEM was removed, and the HepG2 cells were fixed on the coverslip where they were cultured and washed with 1x PBS, and the positive damage control was placed with 250 *μ*M of 30% hydrogen peroxide for 15 min. The SYTOX^®^ Green staining solution was prepared by diluting the stock solution to 1 : 30,000 (167 nm) in 1x PBS; 500 *μ*L of the staining solution was used to cover the cells, followed by incubation for 17 min at 37°C in the dark. The cells were then washed with 1x PBS, the coverslips were mounted with a glycerol gel, and the images were analyzed using a fluorescence inverted microscope, Carl Zeiss 398 Axiovert 40CFL microscope (Carl Zeiss AG, Germany), at a magnification of 200x, with a maximum emission of 504–523 nm.

### 2.7. Acridine Orange (AO) Staining

To prepare the AO staining solution, the stock was prepared by dissolving 1 g of AO in 100 mL of 1x PBS (pH 7.4) that was stored at 4°C away from light. HepG2 cells (10^5^), which were treated with doxazosin and carvedilol, with or without curcumin (1 *μ*M) pretreatment, were stained with 1 *μ*g/mL AO. After incubation for 15 min at 37°C, the stained cells were washed with 1x PBS. The presence of orange marks due to possible cell damage was evaluated using an inverted fluorescence microscope (Carl Zeiss 398 Axiovert 40CFL Microscope; Carl Zeiss AG, Germany) at a magnification of 200x, with a maximum emission of 490 nm in red and 515 nm in green.

### 2.8. Oxidative Stress Detection

Cells were incubated with dihydroethidium (DHE, #D11347, Thermo Fisher Scientific) at 5 *μ*M for 15 min (ex. 490, em. 570 nm), harvested with trypsin, and resuspended in 1x PBS. Finally, cells were evaluated by flow cytometry (Muse Cell Analyzer, Millipore Sigma).

### 2.9. Isolation of Total RNA and Reverse Transcription-Quantitative Polymerase Chain Reaction (RT-qPCR)

Twenty-four hours after treatment with doxazosin and carvedilol, with or without curcumin (1 *μ*M) pretreatment, the cells (10^7^ per treatment) were processed with the Direct-zol™ RNA MiniPrep*^®^* kit, following the manufacturer's protocol. Total RNA was quantified with a Biodrop (Isogen Life Science, Barcelona, España) and stored at −80°C until use. Reverse transcription was performed with 1 *μ*g of total RNA using the RevertAid First-Strand cDNA Synthesis Kit (K1621, Thermo Scientific, Waltham, MA, USA). Subsequently, real-time qPCR was performed using the Maxima SYBR Green/ROX qPCR Master Mix (2x) (K0221, Thermo Scientific, Waltham, MA, USA) on a StepOne apparatus (Applied Biosystems) with the following conditions: 50°C for 2 min, 95°C for 3 min, 40 cycles of 95°C for 45 s, and 60°C for 45 s. The oligonucleotides used are shown in Table 1. Relative expression levels were normalized against 18S rRNA as an internal housekeeping gene, and differences were determined by employing the *ΔΔ*Ct relative method.

### 2.10. Statistical Analysis

Statistical analyses were performed using Microsoft Excel and GraphPad Prism 6. The results are presented as mean ± SD. D'Agostino-Pearson's normality test, analysis of variance (ANOVA), and Dunnett's post hoc test were used for multiple comparisons. *p* values less than 0.05 were considered statistically significant and are indicated by asterisks as follows: ^∗^*p* < 0.05, ^∗∗^*p* < 0.01, ^∗∗∗^*p* < 0.001, and ^∗∗∗∗^*p* < 0.0001.

## 3. Results

### 3.1. Cytotoxicity Assessment Using MTT Assay

**Figure 1:**
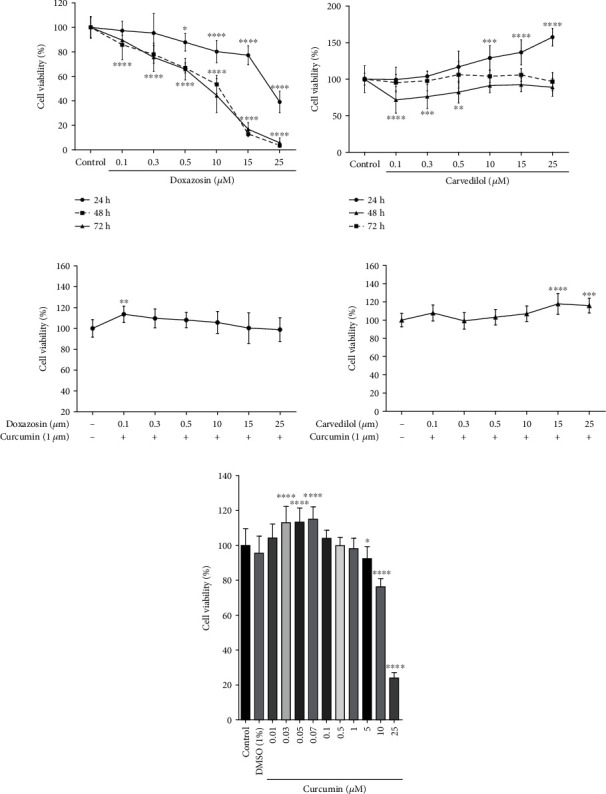
Cytotoxic activities of doxazosin, carvedilol, and curcumin on HepG2 cells. (a) Doxazosin reduces cell viability timeline and dose dependent in the HepG2 cell line. (b) Carvedilol induces proliferation of HepG2 cells at 24 h, while at 48 h, carvedilol is cytotoxic at low concentrations. (c) Curcumin protects HepG2 cells against the induced cytotoxicity for doxazosin. (d) Curcumin pretreatment-maintained viability in HepG2 cells treated with carvedilol. (e) Curcumin hormesis in HepG2 cells, the viability was analyzed by the MTT assay for 24 h and is presented relative to the activity at the start of the experiment in each case. The results are from three independent experiments. Data are mean ± SD; ^∗^*p* < 0.05, ^∗∗^*p* < 0.0025, ^∗∗∗^*p* < 0.001, and ^∗∗∗∗^*p* < 0.0001 versus the control.

Growth inhibitory activity was evaluated using the MTT assay. Cell viability was observed following treatment with different doses of doxazosin and carvedilol for 24, 48, and 72 h. The cell viability was reduced following treatment with doxazosin, which induced a time- and dose-dependent cytotoxic effect. After 24 h treatment with 0.5 *μ*M doxazosin, cell viability was lowered to 87.81% ± 3.95%. After 48 h treatment with 0.1 *μ*M doxazosin, cell viability was lowered to 85.81% ± 3.53% (Figure 1(a)).

On the other hand, cells were slightly less sensitive to carvedilol and showed normal viability after 24 h treatment with 0.1, 0.3, and 0.5 *μ*M of carvedilol, without differences compared to the control (viability 96.54% ± 3.62%). However, 10 *μ*M carvedilol increased HepG2 cell proliferation (cell viability 129.0% ± 4.887%). In contrast, 48 h treatment with low concentrations (0.1, 0.3, and 0.5 M) of carvedilol was found to be cytotoxic (Figure 1(b)).

### 3.2. Cell Viability of Curcumin Treatment in HepG2 Cells

The MTT assay showed that curcumin at low doses (< 1 *μ*M) did not show cytotoxic activity. However, doses of 10 and 25 *μ*M induced cell death (viability 76.33% ± 1.097% and 24.01% ± 0.72%, respectively). These results confirmed that 1 *μ*M curcumin had no toxic effect on cell viability (98.18% ± 1.39%) and was selected as a standard pretreatment for subsequent experiments involving treatment with doxazosin and carvedilol (Figure 1(e)).

### 3.3. Curcumin Reduced Cytotoxicity Induced by Doxazosin and Carvedilol

Treatment with 1 *μ*M curcumin for 1 h before exposure to the antagonists reduced cytotoxicity in HepG2 cells. The viability of curcumin-pretreated cells exposed to 25 *μ*M doxazosin and 25 *μ*M carvedilol (higher dose in both treatments) was increased to 98.83% ± 3.28% and 115.9% ± 2.33%, respectively, compared to the control (Figures 1(c) and 1(d)).

### 3.4. Effects of Doxazosin and Carvedilol on Monolayer of HepG2 Cells

**Figure 2:**
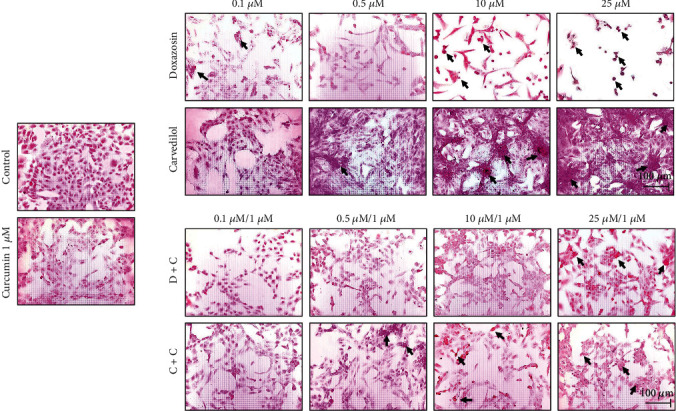
Morphological effects on the monolayer of HepG2 cells exposed to *α-* and *β*-adrenoblockers. The arrows indicate the cells' main morphological changes as alteration of the monolayer, decreased cell interaction, aggregates of eosinophilic cells, and balonization. Curcumin protects the integrity of the monolayer during an interaction with proven antifibrotic drugs. Curcumin decreases the amount of cell death, maintains the integrity of the monolayer, increases the interaction between cells, reduces cell aggregates, and decreases balonization (arrow). Treatments for 24 h were visualized by hematoxylin and eosin staining taken at 200x of magnification.

The morphological changes in the monolayer of HepG2 cells after exposure to doxazosin and carvedilol for 24 h were described as a decrease in the interaction between the cells compared to the control. Doxazosin treatment resulted in dose-dependent balonization and general damage at the monolayer detached from adjacent hepatocytes (Figure 2). Treatment with 25 *μ*M carvedilol generated aggregates of eosinophilic cells and cell conglomerates (Figure 2). However, after pretreatment with 1 *μ*M curcumin, cell viability and morphology were found to be maintained during interaction with doxazosin (doxazosin+curcumin (D+C)) and carvedilol (carvedilol+curcumin (C+C)). In addition, cell death decreased, the monolayer integrity was maintained due to an increased interaction between the cells, fewer cell aggregates were visualized, and the number of balonized cells was decreased (Figure 2).

### 3.5. Ultrastructural Effects of Doxazosin and Carvedilol on HepG2 Cells and Protection of Cell Integrity Generated by Curcumin

**Figure 3:**
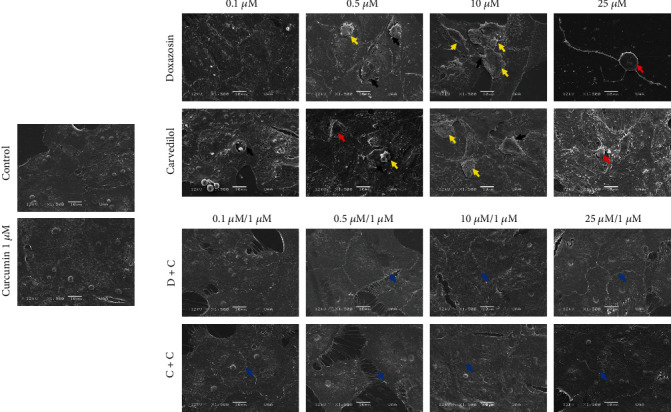
Ultrastructural effects of doxazosin and carvedilol on HepG2 cells. Black arrow: irregular cell surface with folds of the cell membrane. Yellow arrow: cell contraction with the formation of possible apoptotic bodies followed by fragmentation. Red arrow: balonization resulting in separation of neighboring cells. Curcumin reduces the morphological variations related to cell damage caused by *α-* and *β*-adrenoceptor blocking drugs. Cell morphology shows normal villi and intercellular junctions (blue arrow), without possible apoptotic bodies formation in HepG2 cells. Treatments for 24 h were visualized under SEM (5 *μ*M scale bar).

HepG2 cells showed proapoptotic time-and dose-dependent changes with treatment of doxazosin and carvedilol. At 24 h posttreatment, several proapoptotic cells (exhibiting cell shrinkage morphology) as well as cells with cytoplasmic contraction, nuclear condensation, and shedding among adjacent cells were observed. Cells exposed to doxazosin and carvedilol doses ≥ 10 *μ*M presented features such as balonization or cell rounding, condensation of cytoplasmic organelles, the appearance of irregularities on the cell surface, cell fragmentation, and the appearance of apoptotic bodies (Figure 3). However, when cells were exposed to curcumin (1 *μ*M) before treatment with the *α-* and *β*-adrenoblockers, either doxazosin+curcumin (D+C) or carvedilol+curcumin (C+C), the morphology did not show differences between the treated and control cells and presented a normal nucleus, indicating the no onset of apoptosis or necrosis (Figure 3).

### 3.6. Curcumin Reduced Membrane Permeability in HepG2 Cells Induced by Doxazosin and Carvedilol

**Figure 4:**
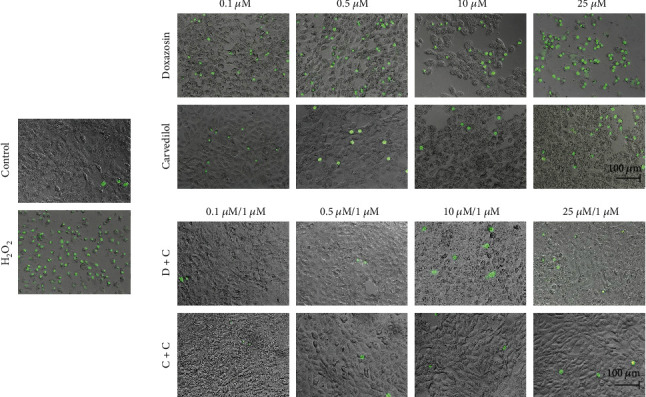
Determination of necrosis marker by fluorescence microscopy of SYTOX green following doxazosin and carvedilol treatment. Doxazosin shows dose-dependent changes in the number of necrotic cells for 24 h. Carvedilol exhibit a lower number of cells dead related to the positive control for necrosis (HepG2 cells exposed to 250 *μ*M hydrogen peroxide for 15 min). Pretreatment with curcumin maintains the viability of the cells because the staining is impermeant for apoptotic and live cells. Staining taken at 200x of magnification.

SYTOX green staining shows a distinction between dying cells with intact plasma membrane integrity and necrotic cells. The dose-dependent effect of doxazosin and carvedilol was apparent by staining of dead cells with intense green fluorescence resulting from binding of SYTOX green to cellular nucleic acids. The damage caused by 25 *μ*M doxazosin was very similar to that observed in the H_2_O_2_ control. However, pretreatment with 1 *μ*M curcumin before applying doxazosin (D+C) or carvedilol (C+C) reduced the numbers of stained cells and necrotic cells, with no difference compared to the control (Figure 4).

### 3.7. Doxazosin- and Carvedilol-Induced Cell Damage and Curcumin-Generated Protection in HepG2 Cells

**Figure 5:**
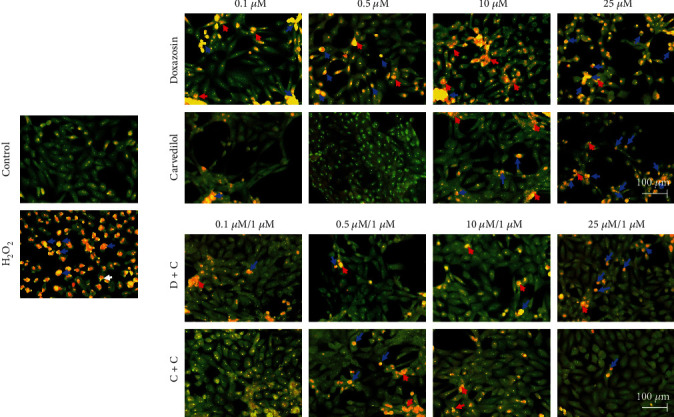
Curcumin protects HepG2 cells from the possible proapoptotic effect of doxazosin and carvedilol. HepG2 cells treated with doxazosin and carvedilol showed morphological changes characteristic of apoptosis: nucleus fragmentation (red arrow) and formation of possible apoptotic bodies (blue arrow). However, the pretreatment with curcumin decreases damage in the cell line compared to the control (untreated cells), which was stained bright green. Treatments for 24 h were visualized by acridine orange staining (at 200x of magnification**).**

The morphological characteristics of the apoptotic cells induced by doxazosin and carvedilol were determined using AO staining. Doxazosin and carvedilol treatment of cells for 24 h induced several morphological changes such as apoptosis-like cell shrinkage, nuclear fragmentation (red arrow), and formation of apoptotic bodies (blue arrow) compared to the control cells or cells treated with curcumin that showed normal morphology. Curcumin has a protective effect against the possible proapoptotic effects of doxazosin and carvedilol. Therefore, treatment with curcumin decreased the number of apoptotic bodies and increased the number of cells with nuclear condensation. Furthermore, the cells showed a dose-dependent effect with curcumin treatment at different doses, like the control cells (Figure 5).

### 3.8. Oxidative Stress Induced by Doxazosin and Carvedilol Decreases in Response to Curcumin

**Figure 6:**
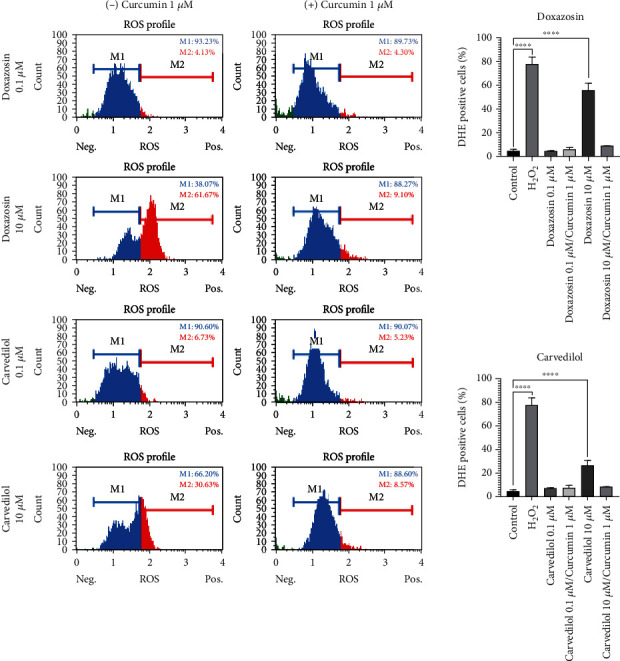
Oxidative stress induced by doxazosin and carvedilol. HepG2 cells were pretreated with the antioxidant curcumin (1 *μ*M) 1 h before doxazosin and carvedilol (0.1 and 10 *μ*M) was added, and cells were analyzed 24 h after treatment. Doxazosin and carvedilol (10 *μ*M) induced a significant increase in ROS production compared to 0.1 *μ*M concentration that did not increase the level of ROS. The antioxidant curcumin pretreatment decreased ROS levels induced by doxazosin and carvedilol. Hydrogen peroxide (H_2_O_2_, 20 mM for 15 min) was used as the positive control of oxidative stress. Flow cytometry analysis of treated cells stained with DHE. Histograms are representative of 3 independent experiments. Statistical analysis of the flow cytometry analysis. A probability value of ^∗^*p* < 0.05 was considered statistically significant.

Doxazosin induced oxidative stress and possible cell death. HepG2 cells were exposed to hydrogen peroxide (H_2_O_2_) for 15 min as a positive control of oxidative stress (Figure 6). Oxidative stress was induced by doxazosin and carvedilol before exposure to curcumin and was analyzed at 24 h using DHE. Untreated and pretreated cells with curcumin showed low levels of oxidative stress, while doxazosin and carvedilol (10 *μ*M) induced a significant increase in ROS production compared to 0.1 *μ*M concentration that did not increase the level of ROS (Figure 6). The antioxidant curcumin pretreatment decreased ROS levels induced by doxazosin and carvedilol. Positive cells for ROS increased to 61% in response to doxazosin 10 *μ*M and decreased to 9% when curcumin was applied before the treatment (Figure 6).

### 3.9. Curcumin Increased the Expression of *Bcl-2* and *Nrf2* in Cells Treated with Doxazosin and Carvedilol

**Figure 7:**
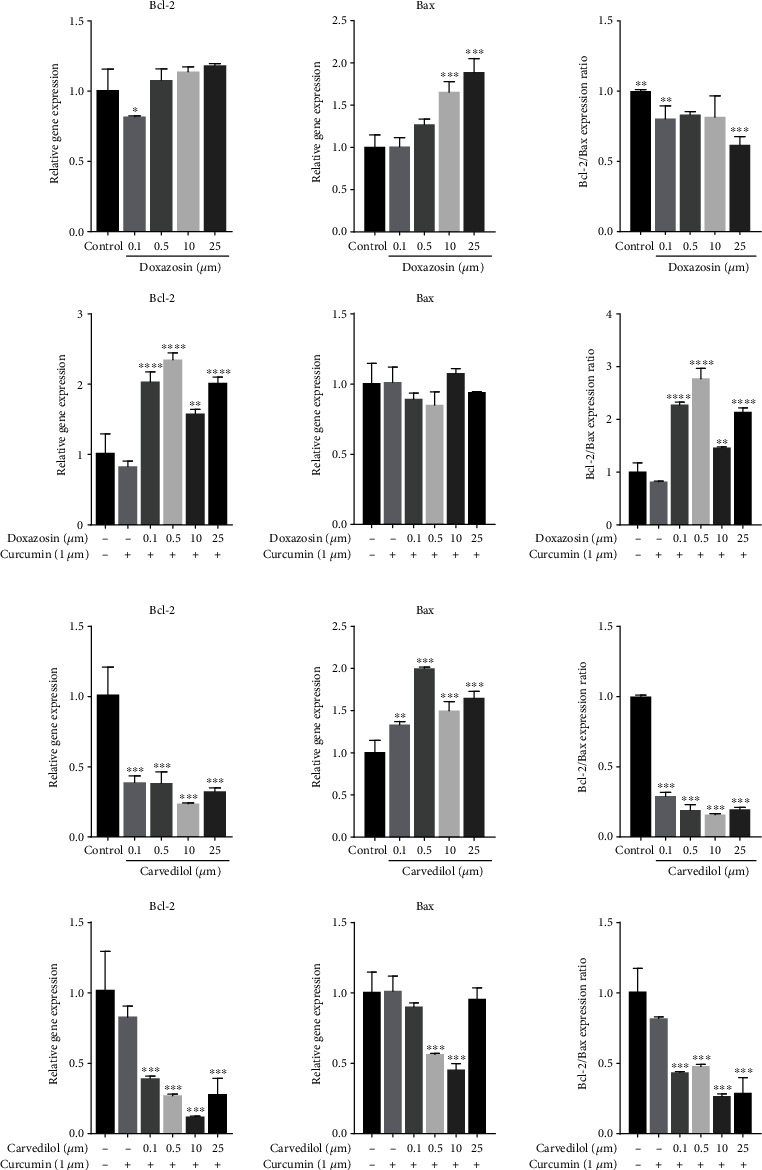
RT-qPCR analysis for *Bcl*-2 mRNA expression, *Bax* mRNA expression, and the Ratio of *Bcl-2/Bax* after treatment with doxazosin and carvedilol at 24 h. (a) Doxazosin upregulated the proapoptotic gene *Bax*, over the antiapoptotic, *Bcl*-2, with the doses of doxazosin treatment mainly at 10 and 25 *μ*M. Interestingly, the *Bcl-2/Bax* expression ratio increased with the treatment curcumin. (b) Carvedilol represents the downregulation of *Bcl*-2 and upregulation of *Bax*. The graphic shows the *Bcl-2/Bax* expression ratio with a value lower than 1. Curcumin pretreatment regulates the propoptotic expression of *Bax*, and the graphic shows the *Bcl-2/Bax* expression ratio with a value higher than 1. The results are from three independent experiments. Data are mean ± SD; ^∗^*p* < 0.05, ^∗∗^*p* < 0.0025, ^∗∗∗^*p* < 0.001, and ^∗∗∗∗^*p* < 0.0001 versus the control.

Statistically significant differences were observed in the fold change of the mRNA expression levels of *Bax, Bcl-2*, *Nrf2*, *Keap*1, *SOD*, and *HO-1* in HepG2 cells treated with doxazosin and carvedilol, with or without curcumin pretreatment, compared with the untreated cells, using RT-qPCR (*p* < 0.05). In particular, 10 and 25 *μ*M doxazosin upregulated the proapoptotic gene *Bax* and downregulated the antiapoptotic gene *Bcl-2* expression. Interestingly, the *Bcl-2*/*Bax* expression ratio was increased in cells subjected to curcumin pretreatment (Figure 7(a)). These results strongly support apoptotic induction by doxazosin through upregulation of *Bax* along with the downregulation of *Bcl-2* mRNA expression. Carvedilol treatment led to the downregulation of *Bcl-2* and upregulation of *Bax* expression (Figure 7(b)). The graph shows that the *Bcl-2/Bax* expression ratio is lower than untreated cells. Therefore, curcumin pretreatment regulates the proapoptotic expression of *Bax* and the *Bcl-2/Bax* expression ratio is higher than untreated cells.

**Figure 8:**
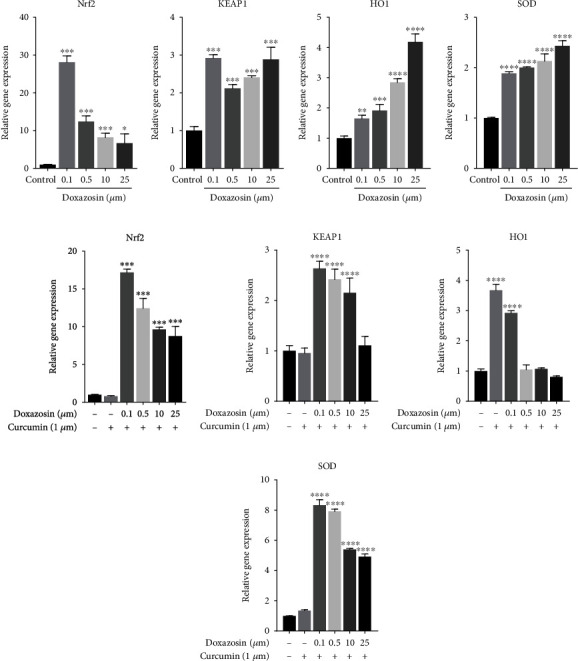
RT-qPCR analysis for *Nrf2*, *Keap1*, *HO-1*, and *SOD* mRNA expressions after treatment with doxazosin at 24 h in the presence and absence of curcumin. The mRNA expression of *Nrf2* were increased by doxazosin treatment (a,e) in the presence and absence of curcumin followed by increase in *Keap1* expression compared to the control group (b,f). The increase of *Nrf2* in cells treated with doxazosin caused the increased of *HO-1* and *SOD* (c,d) while the effect of cotreatment with curcumin regulated the expression of these genes (g,h), possibly due to the control of the oxidative stress generated. The results are from three independent experiments. Data are mean ± SD; ^∗^*p* < 0.05, ^∗∗^*p* < 0.0025, ^∗∗∗^*p* < 0.001, and ^∗∗∗∗^*p* < 0.0001 versus the control.

**Figure 9:**
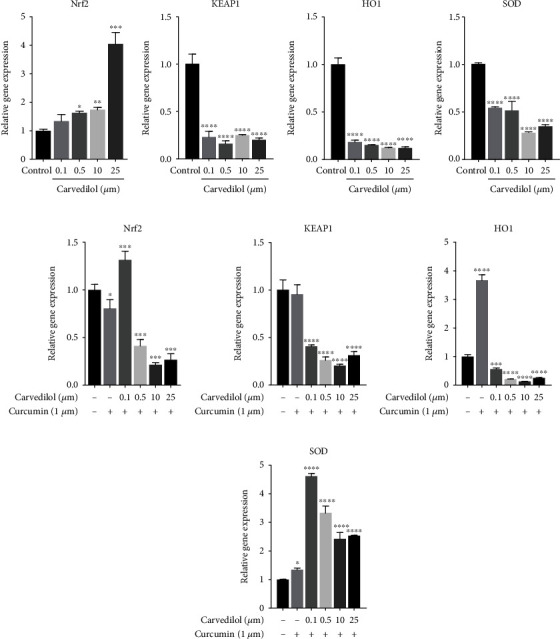
RT-qPCR analysis for *Nrf2*, *Keap1*, *HO-1*, and *SOD* mRNA expressions after treatment with carvedilol at 24 h. Carvedilol shows mRNA expression levels of *Nrf2* upregulated compared to the control with the dose 25 *μ*M and a decrease in *Keap1* compared to the control group in the presence and absence of curcumin (a,b,e,f). Curcumin pretreatment resulted in the activation of the *Nrf2* gene and as result increased the antioxidant gene expression of *HO-1* and *SOD* after the treatment (c,d,g,h). Data are expressed as the mean ± SD (*n* = 3). The results are from three independent experiments. Data are mean ± SD; ^∗^*p* < 0.05, ^∗∗^*p* < 0.0025, ^∗∗∗^*p* < 0.001, and ^∗∗∗∗^*p* < 0.0001 versus the control.

The mRNA expression of *Nrf2* was increased by doxazosin treatment in the presence and absence of curcumin, followed by increase in *Keap*1 expression, compared to the control group. The most significant increases in the expression of *Nrf2* and *Keap*1 were observed at a concentration of 0.1 *μ*M (Figures 8(a), 8(b), 8(e), and 8(f)). With a concomitant increase in *Nrf2* expression in cells treated with doxazosin, an increase in *HO-1* and *SOD* expression was also observed (Figures 8(c) and 8(d)), while in cells treated with doxazosin and curcumin, the expression levels of *HO-1* and *SOD* increased with 0.1 *μ*M doxazosin treatment and subsequently decreased with treatment at higher concentrations of doxazosin (Figures 8(g) and 8(h)). At a dose of 25 *μ*M, carvedilol treatment upregulated the mRNA expression of *Nrf2* (Figure 9(a)), decreased *Keap*1 (Figure 9(b)) and *HO-1* (Figure 9(c)) expressions, and increased *SOD* expression (Figure 9(d)), compared to the control group. Curcumin treatment along with carvedilol has a similar effect to that observed with doxazosin plus curcumin, showing an initial increase in the antioxidant response generated by *Nrf2* and subsequent decrease in expression at higher concentrations of carvedilol (Figures 9(e)–9(h)).

## 4. Discussion

Based on important antecedents of the interaction of doxazosin and carvedilol with the liver parenchyma, several studies have been conducted, wherein these drugs were used to treat liver fibrosis in animal models, and morphological changes and alterations in levels of liver proliferation markers were observed [4, 9–11]. Recently, the morphological changes induced by these drugs in the HepG2 cells have been reported (unpublished data [12]). Based on these results, the present study was developed to analyze the effect of doxazosin or carvedilol on cytotoxicity, morphological changes, mRNA expression of antioxidant response genes, and cell death in HepG2 cells, which was used as a cell model liver. In addition, the present study demonstrates that curcumin acts as an effective protector against cytotoxicity and morphological alterations and regulates the expression of oxidative stress markers in cells treated with doxazosin and carvedilol. Doxazosin showed time-dependent cytotoxicity after 24 h of exposure, even at lower concentrations. However, carvedilol exhibited a proliferation effect in cells that was significantly different at 24, 48, and 72 h.

Doxazosin and carvedilol caused morphological changes in HepG2 cells, and the treated cells were distinguished from untreated cells by their round shape or balonization. Serna-Salas et al. [4] demonstrated the effect of these drugs in hepatocytes through H&E staining and observed similar morphological changes in the in vivo model. Therefore, the administration of doxazosin and carvedilol in HepG2 cells could cause oxidative stress and apoptosis or cell necrosis. According to H&E staining results, doxazosin induced a decrease in cell interaction and balonization, and carvedilol induced the formation of eosinophilic aggregates at higher doses, as observed in chronic lesions in in vivo liver fibrosis models [4, 13]. Curcumin maintained the integrity of the monolayer, during the interaction with doxazosin and carvedilol, possibly due to a decrease in cellular damage caused by these drugs. The effect of curcumin on the cell monolayer was also observed in liver samples obtained from an animal model of liver injury treated with doxazosin and curcumin, as a better recovery of the liver parenchyma in animals treated with curcumin was observed [9]. To determine whether treatments with doxazosin and carvedilol generated HepG2 cell membrane permeability, SYTOX green staining was performed, which allows distinguishing living cells from dead cells [14]. Doxazosin caused dose-dependent damage to the plasma membrane of cells, while carvedilol showed fewer positive cells for this assay. However, treatment with curcumin reduced the membrane damage caused by doxazosin and carvedilol at all doses, consistent with the results of SYTOX green staining. Curcumin can exert protective effects by acting as an antioxidant; this activity can be attributed to its structure, bis-*α*, -unsaturated 𝛽-diketone. Furthermore, curcumin can modify thiol *Keap*1 clusters to release *Nrf2* that migrates to the nucleus and induces the expression of antioxidant enzymes [15–17]. A study on liver cells treated with cycloheximide describes the morphological characteristics of hepatocytes in a state of apoptosis by means of transmission electron microscopy; the observed features included condensation of the cytoplasm, nuclear pyknosis, cell contraction, and cell fragmentation [18]. Consistent with these reports, in our study, we found similar morphological alterations in HepG2 cells treated with doxazosin and carvedilol that may correspond to the process of apoptosis. In addition, we observed multiple fluorescent zones in cells treated with doxazosin, suggesting a progressive fragmentation of the cell body, after which the resultant fragments may give rise to apoptotic bodies.

To analyze whether the morphological changes in HepG2 cells treated with doxazosin and carvedilol corresponded to an apoptosis process and the cells were possibly undergoing drug-activated oxidative stress, we evaluated expression of apoptosis and oxidative stress markers, including *Bax*, *Bcl-2*, *Nrf2*, *Keap*1, *HO-1*, and *SOD*. The 18S rRNA gene was used as an internal control for real-time PCR analysis because it constitutes up to 80%–90% of total cellular RNA [19].

The Bcl-2 family of proteins is the central regulator of the cell-intrinsic apoptotic pathway. Bcl-2 binds to proapoptotic members, such as *Bax*, preventing pore formation and cytochrome c release. In contrast, an increase in the expression of *Bax* induces cell death [20, 21]. Therefore, an altered ratio of proapoptotic and antiapoptotic Bcl-2 family members might be important for understanding the sensitizing effect of doxazosin and carvedilol in these cells. Doxazosin has been reported to induce apoptosis in prostate cells and fibroblasts to treat benign prostatic hyperplasia [22]; the structural quinazoline ring of doxazosin may possibly mediate this effect [23].

Furthermore, in this study, the induction of apoptosis by the highest concentrations of doxazosin was possibly activated by oxidative stress. The results demonstrate that the effects of doxazosin and carvedilol on apoptosis induction in HepG2 cells significantly increased in a dose-dependent manner. When HepG2 cells were treated with doxazosin and carvedilol for 24 h, *Bax* and *Bcl-2* mRNA expression was markedly detectable, but the ratio of expression *Bcl-2/Bax* was low suggesting a proportional increase of *Bax* over *Bcl-*2. It is known that *Bax* and caspase-9 mRNA expression is also upregulated by the remaining p53 in cells with oxidative stress damage [24, 25]. Several studies have shown that cytoplasmic p53 is associated with mitochondria-mediated caspases. p53 settles in the mitochondrial membrane after an importation step involving the mortalin–p53 complex. Similar conclusions were drawn from the results of AO staining, where multiple orange-stained areas were observed in the cytoplasm of cells treated with these drugs.

Furthermore, the results showed an increase in *HO-1* and *SOD* activity after treatment with doxazosin and carvedilol, due to damage generated by oxidative stress. However, carvedilol prevented the increase in *HO-1* activity, probably due to the antioxidant mode of action of this drug. It has been suggested that carvedilol inhibits lipid peroxidation by scavenging free radicals [26]. In particular, upon cotreatment with doxazosin and curcumin, *Nrf2* mRNA was overexpressed, and it was shown that the expression of the effector mRNA *HO-1* and *SOD* also increased, observing a protective effect of curcumin on HepG2 cells during its interaction with doxazosin. Similar studies have demonstrated that the overexpression of *Nrf2* and antioxidant enzymes (*HO-1* and *SOD*) by curcumin treatment protects against different events of cellular damage due to oxidative stress, such as in models of cardiac ischemia, glomerulonephritis, and neuronal damage [15, 27, 22]. Therefore, curcumin pretreatment tends to activate the detoxification signaling pathway genes responsible for protection that are under the control of genes called vitagenes. Vitagenes are a set of genes that are responsible for maintaining cellular homeostasis during stress [28]. The transcription factor *Nrf2* upon binding to the ARE element in the nucleus encodes phase II proteins and antioxidant enzymes such as *HO-1*, Hsp70, thioredoxin, and thioredoxin reductase [29].

Curcumin treatment modulates *Nrf2* expression and exerts antioxidant effects by upregulating the expression of *HO-1* and SOD [27]. In line with these data, our results showed that upon pretreatment with curcumin and subsequent exposure to doxazosin or carvedilol at different doses, the expression of *HO-1* and *SOD* in the cells was regulated. It has been reported that *Nrf2* binds to the promoter region of ARE in the nucleus at the 5′-flanking region and promotes the activation of expression of several phase II detoxification and antioxidant genes [29, 30]. The activation of gene expression occurs through the binding of *Nrf2* to the ARE region with high affinity. Activation of the *Keap*1/*Nrf2* signaling pathway also leads to the induction of expression of antioxidant enzymes, such as GPx, SOD, glutathione reductase, and GSH, all of which can scavenge xenobiotics [31]. Our gene expression data suggest that curcumin mediates the disruption of *Keap*1 dimerization and triggers the release of *Nrf2* by protecting HepG2 cells through the expression of *HO-1* and *SOD*. However, a decline in the expression of *HO-1* and *SOD* was observed during 24 h treatment with carvedilol and doxazosin after curcumin pretreatment, possibly because curcumin can activate SIRT1 signaling, which reduces mitochondrial damage. SIRT1 reduces the levels of molecules such as SOD, succinate dehydrogenase, cytochrome c oxidase, aldehyde methane dicarboxylic acid, and H_2_O_2_ in the mitochondria [30, 31].

## 5. Conclusions

Curcumin protects HepG2 cells against oxidative stress-induced cell damage by scavenging ROS through activation of antioxidant genes in the cells, thereby reducing cytotoxicity, reversing the morphological changes induced by the *α-* and *β*-adrenoblockers, and attenuating secondary effects. Therefore, it could serve as a treatment for the regeneration process of liver parenchyma in the regression of fibrosis with the *α*-adrenoblocker doxazosin and the *β*-adrenoblocker carvedilol.

## Figures and Tables

**Table 1:** Forward and reverse primer PCR sequences for real-time PCR.

Target gene	Forward 5′-3′	Reverse 5′-3′
*Bcl-*2	GACTTCGCCGAGATGTCCAG	GAACTCAAAGAAGGCCACAATC
*Bax*	CGAACTGGACAGTAACATGGAG	CAGTTTGCTGGCAAAGTAGAAA
*Nrf2*	AATGAGCTATTGGCAAGGTACC	CTCTTCAGGAGAGTAGCTGTTG
*Keap*1	ATTCAGCTGAGTGTTACTACCC	CAGCATAGATACAGTTGTGCAG
*HO-1*	CCTCCCTGTACCACATCTATGT	GCTCTTCTGGGAAGTAGACAG
*SOD*1	ATCCTCTATCCAGAAAACACGG	GCGTTTCCTGTCTTTGTACTTT
18srRNA	AAACGGCTACCACATCCAAG	CCTCCAATGGATCCTCGTTA

## Data Availability

The datasets used and/or analyzed during the current study are available from the corresponding author on reasonable request.

## Abbreviations

*α*: Alpha
*β*: Beta
*μ*L: Microliter
AO: Acridine orange
CC_I4_: Carbon tetrachloride
CO_2_: Carbon dioxide
DMSO: Dimethyl sulfoxide
DMEM: Medio Dulbecco's modified Eagle's medium-low glucose
H_2_O_2_: Hydrogen peroxide
MTT: (3-(4,5-Dimethylthiazolyl-2)-2,5-diphenyltetrazolium bromide)
*Nrf2*: Nuclear factor erythroid-derived 2-like 2
*HO-1*: Heme oxygenase-1
*SOD1*: Superoxide dismutases 1
*Bax*: Bcl-2-associated X protein
BCL-2: *B-cell lymphoma 2*
ROS: Reactive oxygen species
SEM: Scanning electron microscope
*Keap*1: Kelch-like ECH-associated protein 1.

## Additional Points

*Highlights.* (i) Curcumin generates protection against oxidative stress in liver cells treated with two experimental drugs (doxazosin or carvedilol) used to decrease liver fibrosis. (ii) Curcumin controls doxazosin- and carvedilol-induced cytotoxicity and morphological changes in HepG2 cells. (iii) The Bcl-2 relationship on *Bax* was higher in cells treated with curcumin before doxazosin or carvedilol. (iv) Curcumin protects HepG2 cells against oxidative stress-induced cell damage by scavenging ROS through overexpression of Nrf2.
